# Implementation of evidence-based health promotion and disease prevention interventions: theoretical and practical implications of the concept of transferability for decision-making and the transfer process

**DOI:** 10.1007/s00103-021-03324-x

**Published:** 2021-04-23

**Authors:** Tamara Schloemer, Freia De Bock, Peter Schröder-Bäck

**Affiliations:** 1 Department of International Health, Faculty of Health, Medicine and Life Sciences, Care and Public Health Research Institute (CAPHRI), Maastricht University, PO Box 616, 6200 MD Maastricht, Limburg The Netherlands; 2 Head of Division “Effectiveness and Efficiency of Health Education”, Federal Centre for Health Education (BZgA), Cologne, North Rhine-Westphalia Germany; 3 Department of Applied Health Sciences, Hochschule für Gesundheit, Bochum, North Rhine-Westphalia Germany; 4 University of Applied Sciences for Police and Public Administration in North Rhine-Westphalia (HSPV NRW), Campus Aachen, Aachen, Germany

**Keywords:** Public Health, Implementation Science, Effectiveness, Evaluation, Complex intervention, Public Health, Implementierungswissenschaften, Effektivität, Evaluation, Komplexe Intervention

## Abstract

Evidence-based health promotion and disease prevention require incorporating evidence of the effectiveness of interventions into policy and practice. With the entry into force of the German Act to Strengthen Health Promotion and Prevention (PrävG), interventions that take place in people’s everyday living environments have gained in importance. Decision-makers need to assess whether an evidence-based intervention is transferable to their specific target context. The Federal Centre for Health Education (BZgA) recommends that transferability of an intervention should be clarified before any decision to implement it. Furthermore, transferability needs to be finally determined after an evaluation in the target context. In this article, we elaborate on theoretical and practical implications of the concept of transferability for health promotion and disease prevention based on the Population–Intervention–Environment–Transfer Models of Transferability (PIET-T). We discuss how decision-makers can anticipate transferability prior to the intervention transfer with the help of transferability criteria and how they can take transferability into account in the further process. This includes the steps of the analysis of a health problem and identification of effective interventions, the steps of the initial transferability assessment and identification of the need for adaptation, and the steps of the implementation and evaluation. Considering transferability is a complex task that comes with challenges. But it offers opportunities to select a suitable intervention for a target context and, in the transfer process, to understand the conditions under which the intervention works in this context. This knowledge helps to establish an evidence base, which is practically relevant.

## Abstract (de)

Evidenzbasierte Gesundheitsförderung und Prävention erfordern die Einbettung effektiver Interventionen in Politik und Praxis. Mit dem Inkrafttreten des Präventionsgesetzes (PrävG) haben Interventionen, die in der Lebenswelt der Menschen stattfinden, an Bedeutung gewonnen. Entscheidungsträger müssen beurteilen, ob eine evidenzbasierte Intervention auf ihren spezifischen Zielkontext übertragbar ist. Die Bundeszentrale für gesundheitliche Aufklärung (BZgA) empfiehlt, die Übertragbarkeit vor jeder Entscheidung zur Implementierung einer Intervention zu klären. Darüber hinaus muss die Übertragbarkeit nach einer Evaluation im Zielkontext endgültig bestimmt werden. In diesem Artikel elaborieren wir theoretische und praktische Implikationen des Konzepts der Übertragbarkeit für Gesundheitsförderung und Prävention auf der Grundlage der „Population (P)-Intervention (I)-Environment (E)-Transfer (T) Models of Transferability (PIET-T)“. Wir diskutieren, wie Übertragbarkeit mithilfe von Übertragbarkeitskriterien für die Entscheidungsfindung vor dem Interventionstransfer antizipiert und im weiteren Prozess berücksichtigt werden kann. Dies umfasst die Schritte der Analyse eines Gesundheitsproblems und der Ermittlung wirksamer Interventionen, die Schritte des initialen Assessments der Übertragbarkeit und der Ermittlung des Adaptionsbedarfs sowie die Schritte der Implementierung und Evaluation. Die Betrachtung von Übertragbarkeit ist eine komplexe Aufgabe, die mit Herausforderungen verbunden ist. Sie bietet jedoch das Potenzial, eine geeignete Intervention für den Zielkontext auszuwählen und im Transferprozess die Bedingungen zu verstehen, unter denen die Intervention in diesem Kontext funktioniert. Dieses Wissen hilft, eine Evidenzbasis aufzubauen, die praktisch relevant ist.

## Introduction

The relevance of incorporating evidence of the effectiveness of health promotion and prevention interventions into policy and practice has been acknowledged for decades [1, 2]. In 2015, the German Act to Strengthen Health Promotion and Prevention (Prevention Act [PrävG]) entered into force, assigning far-reaching new responsibilities to the statutory health insurances (Gesetzliche Krankenversicherung [GKV]) for the prevention and reduction of disease risks, the promotion of self-determined health behaviour, and the reduction of social and gender-related health inequalities. The act requires basing interventions on the best available scientific evidence and evaluating their effectiveness in local settings [3, 4]. Evidence-based health promotion and disease prevention are regarded as relevant to reduce supply deficits, such as the waste of limited financial resources [5–7], the disorientation and demotivation of actors and target populations in the absence of intervention effectiveness, and the underestimation of the success of policy measures caused by the implementation of interventions with weak or unknown effectiveness [5]. Furthermore, interventions should not lead to undesirable side effects in predominantly healthy people. The Robert Koch Institute (RKI) [7] demands the selection of health promotion and disease prevention interventions that can actually improve the health of the target population: They must be suitable and effective in the practical implementation on site.

This requirement assumes that the selected interventions are transferable to the target context. Transferability addresses the extent to which an intervention whose effectiveness was established in a primary context is effective in a target context [8–11]. Interventions in health promotion and disease prevention are more or less complex and context dependent. Thus, complexity may refer to the intervention itself (such as components, flexibility, and outcomes), to the intervention context (such as population behaviour or organisational levels targeted), and to the interaction between the two [12–14]. That means, even if an intervention is based on sound scientific evidence, “the intervention itself may be applicable, but it may generate other effects than those seen in the primary intervention” [15, p. 1]. The PrävG focuses on health promotion and disease-prevention interventions in the everyday living environments of the people, i.e., in specific settings such as the community, the school, or the workplace [3, 4]. This focus underscores the importance of anticipating and evaluating transferability of interventions to these specific contexts (see Infobox 1 for an example).

Two essential questions need to be considered: First, with regard to the primary context, what kind of evidence and information is available for decision-makers in order to anticipate transferability? Second, how can transferability be assessed by decision-makers before and during the transfer process? In recent years, few structured approaches have been developed. For example, the first question is addressed by Munthe-Kaas et al. [16], who developed the TRANSFER approach to assist review authors in considering transferability to the context specified in the systematic review in collaboration with stakeholders. Cambon et al. [10] developed a checklist tool (ASTAIRE) with transferability criteria for health promotion interventions. It addresses both questions in that it can be used either to report evidence or to assess transferability to a target context. However, it does not provide guidance for the transfer process itself. Burchett et al. [17] found in their methodological study of the usability and usefulness of assessment tools and frameworks that checklist tools (including transferability tools) have been shown to be insufficient because they fail to address mechanisms on how the intervention works and its potential interactions with the context; these authors express the need to incorporate information on assessment criteria with information on mechanisms of action. Other approaches focus on implementation or evaluation, without systematically considering the concept of transferability and transferability criteria ([13, 18–20]; see Table 1).

**Table 1:** Definitions of transferability and related terms

Adaptation of intervention	“A systematically planned and proactive process of intervention modification with the aim to suit the specific characteristics and needs of a new context and enhance intervention acceptability” [42, p. 9]
Applicability	The feasibility of implementation of an intervention regardless of the outcome(s) [9]
Core elements	Those features in the intent and design of an intervention that fundamentally define the intervention, are responsible for the effectiveness of the intervention, and therefore should not be adapted [42]
Decision-makers	Persons who are involved in decisions on transfer of an intervention to a target context at international, national, regional, or local levels (e.g., politicians, funders, researchers on population health issues, practitioners, and stakeholders who will be affected by an intervention) [43]
Evaluation of outcome	Evaluation of results that provide information about the achievement of the defined intervention’s goal(s)/outcome(s) [12]
Evaluation of process	Evaluation of process data that provide information about what actually happened in the course of the implementation of an intervention in order to document and explain relevant processes. The aim is to hypothetically explain whether and why interventions produce certain results under certain conditions [12]
Generalisability	The perspective of the researcher on generalisability of the results to any context [9]
Intervention fidelity	“The degree to which an intervention is implemented as intended by its developers with the aim to maintain intervention’s intended effects” [42, p. 9]
Primary context	System(s) in which the evidence on a health intervention was established (see Fig. 1; [21])
Target context	System(s) into which the health intervention is transferred (see Fig. 1; [21])
Transferability	The extent to which the outcome(s) of a successful health intervention evaluated in a primary context can be achieved in a target context [9, 11]
Transfer/Implementation	An actively planned and deliberately initiated effort to bring an intervention into policy and practice within a target context [13]

To fill this conceptual gap, Schloemer and Schröder-Bäck [21] developed a theoretical and methodological approach for the assessment of transferability in a qualitative systematic review. In this review, articles on transferability of health interventions were systematically searched, ranked for their quality, and further analysed with a thematic synthesis. This form of an interpretive synthesis shares similarities with approaches from grounded theory and meta-ethnography [22]. Two interrelated models were built through the structured, inductive method on the basis of an identification and systemisation of criteria for transferability: The Population–Intervention–Environment–Transfer Models of Transferability (PIET-T) explain the theoretical concept of transferability and provide guidance to integrate the use of transferability criteria in transfer processes. Based on these models, we aim to discuss theoretical and practical implications of the concept of transferability for health promotion and disease prevention. In the following, we introduce the theoretical understanding of transferability and propose an approach for the integration of information about characteristics of primary and target contexts in transfer processes with consideration of potential practical challenges.

### Theoretical understanding of transferability as a complex concept

**Fig. 1:**
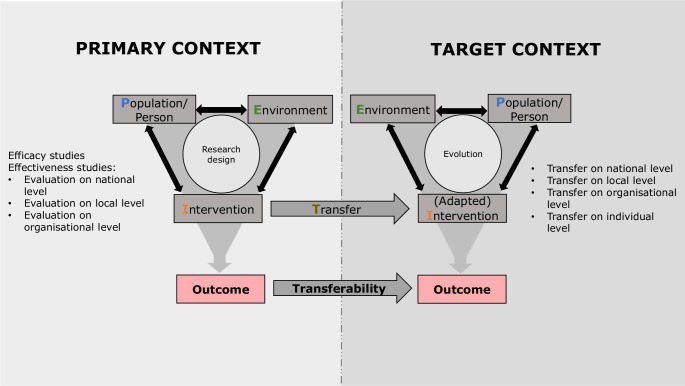
The conceptual Population–Intervention–Environment–Transfer Model of Transferability (PIET-T) shows that the population (*P*), the intervention (*I*), and the environment (*E*) in the primary and target context, as well as the transfer (*T*) itself, influence the transferability of a health intervention [21]

The definition of transferability (see Table 1) does not provide insight into the underlying complexity of this concept. A prerequisite for an assessment of transferability is knowledge of the intervention contexts and conditions [21, 23]. The conceptual PIET‑T model shows that the population (P), the intervention (I), and the environment (E) in the primary and target context, as well as the transfer (T) itself, influence the transferability of a health intervention (see Fig. 1; a comprehensive explanation is available elsewhere [21]). The model serves as a theoretical aid to understand the transferability mechanism for an intervention and may be used before, during, and retrospectively after transfer. For an anticipation of transferability, it addresses the overarching themes (PIET) and the interrelationships that are generally important to consider as a basis for a transfer decision and for process planning. The themes cover specific underlying criteria that may be related to one another and may influence the transferability of an intervention. The model points to the following theoretical key aspects:

- *Transferability* of an intervention is dependent on specific conditions in the primary and target context. For a comparison, information both from the primary context and from the target context is needed on the criteria (factors) proven or thought to be relevant for the success of a specific intervention [11, 21].
- *The primary context* refers to the system(s) with available information on the outcome(s) of one or more studies on an intervention, including the description of the specific study design and processes, and of relevant criteria of the intervention, the study population, and the study environment [21]. For anticipating transferability, it is important to understand how and under which contextual conditions the intervention works and exerts its effects [17, 21].
- *The target context* refers to the system(s) with unique characteristics of the population and the environment into which an intervention is transferred. Depending on the contextual conditions, it may become necessary to adapt the intervention to the target context [9, 10, 17, 21]. The transferred intervention, the population, and the environment of the target context influence one another. An evolution takes place, which addresses the target population and the environment [9, 21, 24, 25]. Depending on the extent of standardisation and flexibility, changes and reactions in the target context may need further adaptation and development of the intervention. In other words, the target context as a system evolving over time needs to be taken into account [21, 26].
- The planning of the* transfer* depends on information obtained from the primary context and the target context. A theoretical distinction is made between effects of the intervention and influences of the process of transfer on the outcome(s) [27, 28]. The transfer of the intervention may have an influence on how the intervention works in the target context. That means that it is relevant as a (more or less changeable) process in the course of evolution because interactions can occur with the intervention and the context. This refers to the time component: The transfer is planned with a specific strategy, but developments during and after implementation should be considered for transferability.
- The *level of transfer* includes considerations of the research designs and the contextual levels in the primary and target context. The information from the primary context can result from one or more efficacy studies (evaluation[s] of an intervention under optimum conditions), or from effectiveness research on one or more contextual levels (evaluation[s] of an intervention when delivered under real-world conditions), or from studies of different research types and contextual levels [21, 29, 30]. The transfer can take place on different contextual levels in a target context, such as the national, local, organisational, and individual levels. The level of transfer from the primary to the target context is relevant for consideration of the comparability of both contexts, such as the transfer from experimental context to real-world context or transfer from one real-world context to another real-world context with similar or different contextual levels and conditions. This also includes transfers from a small scale to a large scale (scale-up) or from one population to another [21, 31].

### Practical implications of considering transferability in health promotion and disease prevention

The concept of transferability has gained in importance for evidence-based health promotion and disease prevention in Germany. In its memorandum, the Federal Centre for Health Education (BZgA) demands that the transferability of an intervention should be clarified before any decision to implement the intervention [32]. When one considers the definition of transferability and key aspects of the conceptual PIET‑T model, the relevance of anticipating transferability for the transfer decision becomes apparent. Furthermore, because transferability aims at the effectiveness of an intervention in a specific target context, it can finally be determined only after an evaluation in the target context [21]. The concept of transferability is, therefore, also related to considerations about the sustainability of an intervention [8, 21, 33].

**Fig. 2:**
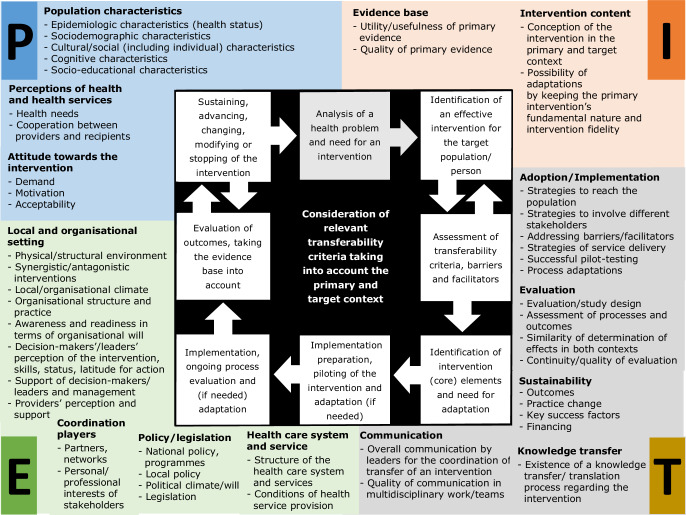
The PIET‑T process model includes descriptive themes and criteria of the population (*P*), intervention (*I*), environment (*E*), and transfer (*T*) and is intended to accompany the steps for determining transferability. The themes and criteria, which are mapped around the process, are intended to help determine which information is relevant for the comparison of primary and target contexts [21]

The PIET‑T process model is intended as a decision-making and planning aid to accompany the steps for determining transferability (see Fig. 2). It includes descriptive (sub-) themes and criteria of the population (P), intervention (I), environment (E), and transfer (T). The themes and criteria are intended to help decision-makers determine which information is relevant for comparison of the primary and the target context. Decision-making can depend on various criteria and conditions. The relevance and use of the criteria can be specific depending on decision-makers, contexts, and interventions. Therefore, the model does not prescribe a rigid review and collection of information for all criteria. Instead, it offers a comprehensive list of potential influences that can be used flexibly for operationalisation according to situational needs and requirements. It may also be used as a theoretical aid in developing documentation and reporting material [21]. Because it forms an overarching framework for the consideration of transferability in decision-making, planning, and transfer, it is open to combination with other instruments. The comprehensive criteria list with examples and a guide for an initial assessment and for process planning are available elsewhere [21].

In the following, we summarise the essential steps of the process model, with consideration of relevant aspects for German health promotion and disease prevention.

## Steps of the analysis of a health problem and identification of effective interventions

A transfer decision should always be based on the need for an intervention [7, 8, 33]. This requires information from the target context. The need can result from the prevalence of a health problem, its public health burden, health priorities and goals, priorities of decision-makers, and the perceived needs of the target population and local actors [8, 33]. This also includes the consideration of existing synergistic or antagonistic interventions, which might influence the need for and transferability of a new intervention [8, 9, 10]. The German RKI [7] calls for the selection of an effective intervention based on the needs determined on site for the target population. Barriers are also reported here, since the relevant data are predominantly available only at the federal or state level. Attempts are therefore increasingly being made to create small-scale health reports that depict the specific health problems in individual municipalities or city districts [7].

In Germany, there is a heterogeneous picture of actors in the field of health promotion and disease prevention. More coordination of measures is recommended, particularly with regard to limited financial resources. Therefore, overarching health goals are formulated, for example in the PrävG [3, 7]. This offers the potential to anticipate and evaluate transferability of interventions to reach these goals in target contexts. The involvement of coordination players (such as networks, partners, and local actors) and the needs and views of the target population in a context may be necessary at an early stage and in all phases of the process [15, 24, 25]. This may facilitate a better understanding of the health problem and potential solutions. Depending on the conditions in the target context, it might be important to clarify who is involved in the determination of specific goals and relevant outcomes for the target population and the subsequent selection of and decision for an intervention (for example, cooperation between researchers, policy makers, local actors, and representatives of the target population) [21]. This also raises the question of the level of participation of relevant stakeholders [7, 34]. These aspects described above are important criteria for the transferability of an intervention, as they might be essential for stakeholders’ acceptance of and involvement in the intervention and, thus, for its sustainability [15, 21, 26, 34]. A helpful instrument from the beginning might be the Criteria for Good Practice in Health Promotion addressing social determinants [35].

The identification of one or more effective interventions with regard to the identified needs and goals is in itself a comprehensive step and may be dependent on many factors, such as available data, resources, knowledge, and experiences of responsible persons. In Germany, the decision to implement health promotion and disease prevention interventions is mostly in the hands of actors from practice or politics. These actors are often not yet familiar with the concept of evidence-based practice [32]. Furthermore, given the variety of prevention and health promotion programmes and interventions available, it can be difficult for a community or organisation to make a choice [7]. A list of relevant databases to use for the search for interventions is available from the BZgA [32].

From the perspective of transferability, two main criteria of the evidence base are important for understanding whether an intervention is appropriate to influence the health problem of the target population: the quality and the usefulness of the primary evidence. Ideally, information on the quality of the evidence is already available in the databases. Based on the consensus statement of the American Society of Prevention Research [29, 36], the BZgA defines standards of evidence for Germany. Accordingly, an intervention is considered to be sufficiently evidence-based if it meets the criteria of efficacy and effectiveness, in particular with regard to (a) a detailed description of the intervention and its pathways in a certain population and a certain context, (b) its effectiveness and safety under everyday conditions, and (c) insights gained from process evaluations for a successful implementation [32]. In addition to collecting information on the quality of the evidence, an equally important and pragmatic approach is assessing the usefulness of the evidence for the target context (see Infobox 2) [21]. If not all criteria of quality and usefulness are met, an intervention can still be transferable. Depending on conditions in the target context, it should be weighed how much loss of quality and usefulness of the evidence can be accepted. In some cases, it may be ethically inacceptable to wait until high-quality evidence from a similar context becomes available [11]—for example, if only the evidence of efficacy is available for an intervention, but the usefulness for the target context is acceptable. On the other hand, if no high-quality evidence is available, a well-described intervention from a practical project evaluated in a similar context might be useful. The selection of one or more potentially suitable interventions builds the basis for the next steps.

## Steps of the initial transferability assessment and identification of the need for adaptation

After an analysis of the need for and identification of an effective intervention, the initial assessment of transferability should compare relevant criteria in both contexts with regard to the population (P) and environment (E) to anticipate conditions that are likely to influence the outcome(s) of the intervention (I) in the target context for decision-making and the planning of the intervention transfer (T) [21] (see Fig. 2). In other words, the assessment should assist decision-makers in systematically analysing what information is available and how to use this knowledge for the transfer decision, intervention implementation, and evaluation of transferability. An explanation and order of criteria use as well as guiding questions for an initial assessment and process planning are available elsewhere [21].

For the transfer decision, it is relevant to discuss whether the relationships between the intervention and the outcome(s) are expected to differ for the population (or subgroups) in the primary and target contexts [21, 23]. The focus should lie on consideration of the conditions under which the intervention is expected to be successful or not in the target context: Is it potentially transferable as intended, is it potentially transferable with adaptations, or is it not transferable at all?

The effort for the anticipation of transferability and decision-making can depend on various aspects, such as the existing data; the complexity of the intervention; and the number, networking, values, and expertise of decision-makers and interventionists or those implementing/delivering the intervention. Furthermore, the number of criteria relevant to analyse might differ with regard to the health problem, the intervention, the compared contexts, and the transfer level. Since basic information from the target context and the primary evidence is ideally already available from the first steps, a possible pragmatic approach is to select criteria that are relevant to assess for decision-making on transferability. Therefore, it may be helpful to note why these criteria are important to consider. Subsequently, it can be determined and documented whether the primary and target contexts differ with regard to the selected criteria, and whether and why this difference is relevant for reaching the expected outcome(s) in the target context. In other words, an emphasis should be placed on criteria that may potentially promote or hinder transferability of the intervention. The aim is to differentiate and select potentially influencing criteria for the further process [21].

For this step, it may be necessary to identify and document what information about criteria is missing and whether it is important to collect additional data for decision-making, as the relevance of detail and validity of information from both contexts may differ among criteria. There may be criteria relevant for the target context that cannot be compared. These should nevertheless be taken into account because data could be collected in the course of the further process, if necessary. In general, conclusions from reviewing evidence for transferability may be limited due to poor description of an intervention [31], a lack of adequate process and contextual information [11, 27], or unknown suitability of an intervention for different contexts [27, 31]. It may be an option to contact intervention developers for collecting information and for advice on the further process, as knowledge exchange between actors in the target context and intervention experts may foster mutual learning and transferability [15, 24, 25, 31, 37].

The validity of information from the target context can be enhanced by gathering data—for example, from epidemiologic and demographic studies in the target context—and by conducting qualitative research with stakeholders [11, 28]. As previously discussed, participatory approaches might be promising, such as in the form of discussions with stakeholders in workshops about the criteria. This may help to uncover facilitators and barriers to the transferability of the intervention, to assess their relevance, and to further specify the relevant potential influences on the outcome(s). Local actors have practical knowledge of the target context. A joint analysis of relevant facilitators and barriers to transferability provides a structured approach to identify the need for adaptations of the intervention or target context. Addressing barriers and needs of professionals before implementing an intervention and enabling adaptations to improve its usability may enhance effectiveness of the intervention [38]. For example, it may be possible to overcome identified barriers with regard to the environment through suitable measures, such as providing resources for intervention delivery. Discussion and identification of an intervention’s core elements and a context-relevant form of the intervention may enable formulation of potentially transferable elements as well as elements that should be adapted in order to facilitate implementation while considering intervention fidelity, and are relevant to planning the evaluation of the intervention’s effects [24, 26, 37]. Such discussions can also reveal whether the intervention is not suitable for the target context. Then the information gathered can be used as a basis for identifying an alternative intervention or developing a new intervention. In the event of subsequent structured adaptations of the intervention, the expanded Framework for Reporting Adaptations and Modifications to Evidence-Based Interventions (FRAME) may be a useful additional tool [39].

## Steps of the implementation and evaluation

When a decision to transfer the intervention has been made, the data from the initial assessment of relevant criteria is a basis for planning the intervention transfer, including implementation preparation, the implementation itself, and evaluation. The transfer criteria (T) are intended to help identify important aspects for transferability with regard to the steps of implementation and evaluation in the target context under consideration of strategies and processes in the primary context. These aspects might be incorporated in an implementation guide or material. The data from the previous steps builds a basis to specify hypotheses and/or questions for the evaluation. Furthermore, transferability criteria relevant for successful implementation and intervention effectiveness can be operationalised to be included in the evaluation of the process (e.g., questions for qualitative analysis) and outcome(s) (e.g., potential moderating factors for quantitative analysis). Piloting the intervention helps to refine hypotheses and implementation and evaluation plans, to pilot adaptations, and to identify the need for further adaptations during the transfer process. Finally, transferability can be explained by measuring whether and under which conditions the intervention is effective in the target context [21].

The choice of evaluation design in the target context can depend on various factors, e.g., ethical, political, or financial reasons [23]. An important aspect is the level of transfer. For health promotion and disease prevention, various authors suggest using phase models as an orientation. The underlying assumption is a sequential fashion of efficacy research, effectiveness research under real-world conditions, and dissemination on a larger scale [2, 23, 30, 31, 40]. Considerations about the evaluation design should ideally be based on the existing primary evidence as well as on changes to the intervention through adaptation in the target context [23, 32]. The BZgA proposes a model with recommendations for Germany: For the transfer of a well-described but hitherto not evaluated intervention from a practical project, an evaluation with a pilot study is recommended; for the transfer of an intervention with plausible prerequisites for effectiveness (“BZgA promising practice”), an effectiveness study with a process evaluation is recommended; and for the transfer of an intervention with causal proof of effectiveness, monitoring during transfer and dissemination is recommended (“BZgA best evidence”). If an intervention with causal proof needs to be adapted, at least a process evaluation should be carried out; in the case of larger adaptations, a new effect evaluation might be needed [32].

Bödeker et al. [23] provide an overview and classification of experimental, quasi-experimental, and nonexperimental study designs for evaluating intervention effects in health promotion and disease prevention, with examples. A structured documentation and ongoing evaluation of the details of implementation processes (by quantitative and qualitative approaches) may elicit facilitators and barriers in the target context and aid in understanding issues of transferability [26]. Depending on the selected outcome(s), the evaluation of transferability goes beyond measuring intervention effectiveness, for example, by investigating return on investment or stakeholder satisfaction. Measurements of the public health impact include reach (participation rate and the representativeness of participants), effectiveness (impact of an intervention on specified outcome criteria, negative outcomes, and intended results), adoption (percentage and representativeness of organisations or settings that conduct the intervention), implementation (intervention fidelity, or the quality and consistency of delivery), and maintenance of the intervention (individual-level long-term results; setting-level institutionalisation of the intervention [RE-AIM]). [2, 20, 38].

Depending on the study design, questions might be answered with regard to the intervention and implementation: Were the hypotheses on transferability confirmed? (For example, was the intervention effective with adaptations?). What are the criteria that contributed to the success of the implementation? What are the lessons learned? (For example, which facilitators and barriers have been identified? Have barriers been removed? How do stakeholders explain the success or failure of the intervention and/or implementation?). Reflections can include a comparison with the primary context. The knowledge reported on transferability can form an information basis for further transfers of the intervention. Reporting statements might be used in addition to transferability criteria, such as the Template for Intervention Description and Replication (TIDieR) checklist and guide [41].

### Infobox 1 Retrospective analysis of transferability by using the published evaluations of the programme “Active health promotion in old age” (“Aktive Gesundheitsförderung im Alter,” “AGil”) [44–50] as an example

The programme “AGil” addresses physical activity, nutrition, and social participation of the elderly. The aim is to strengthen the responsibility of older people (empowerment) and to impart skills for health-promoting behaviours. The intervention (I) includes a half-day advisory event and integrates existing local structures (for example, sports groups) to encourage the realisation of recommendations [45, 46, 48]. It focuses on the following elements: A multidimensional approach (nutrition, physical activity, social participation), an interdisciplinary approach (expert team), a behaviour-oriented approach (didactic concept), and a circumstance-oriented approach (networks) [45].

The programme targets a population (P) with specific epidemiologic, cognitive, and sociodemographic characteristics: independent persons 60 years and older without disabilities or cognitive impairment who live at home [49]. The intervention was developed for and implemented in an urban environmental setting (E) in Hamburg, Germany, in 2000. The effectiveness of the intervention was measured in the Longitudinal Urban Cohort Ageing Study (LUCAS) in Hamburg over a period of 13.8 years [44, 46]. The 1‑year follow-up-evaluation of the intervention (I) in a randomised controlled study showed (inter alia) significant improvements with regard to healthy nutrition and physical activity for participants aged 60 years and older (P). The intervention was transferred (T) from the northern urban area (primary context) to a rural southern area in Baden-Württemberg (Kinzigtal, target context) in 2007 [46, 48, 50].

From the primary context it is known that half of the participants with high functional competence and over a third of participants with low competence had medium or higher education levels. Their culture was shaped by the urban environment [44, 45]. Acceptance of the intervention was high. Relevant environmental characteristics (E) were found in the health care system, which exhibited good conditions of health service provision, providing easy financial and geographic access to the intervention. Multiple coordinating players were involved for intervention delivery in a multiprofessional network [45]. The analysis of the target population (P) aged 60 years and older in Kinzigtal showed some different epidemiologic and sociodemographic characteristics from the population in Hamburg. For example, the participants in Kinzigtal had more functional impairment, and most of them (87%) had a low education level. Their cultural rural lifestyle differed from the urban lifestyle. Although conditions of health service provision (E) were characterised by financial accessibility, geographic accessibility was lower, such as through reduced public transport. The participants came from surrounding villages and small towns [46].

Relevant criteria of transfer (T) for implementation in the target context were the strategies of service delivery (for example, by organising transport to improve accessibility of the intervention), a tailored recruitment strategy, and involvement of different stakeholders. A knowledge transfer was realised through training of providers. For the evaluation, a mixed-methods design was used to measure, inter alia, acceptance and effectiveness of the intervention, as well as implementation barriers and facilitators. The results showed that acceptance of the intervention was high in Kinzigtal, as was seen in Hamburg [46]. The pre-post comparison after 12 months revealed significant changes for healthy nutrition, but not for physical activity [48]. The analysis of barriers and facilitators showed that the didactic concept, the interdisciplinary approach, and also the short duration of the event were facilitators for effectiveness [49]. Barriers were found (inter alia) with regard to the usability of intervention documents for the population (for example, participants had difficulties with writing) and with regard to recommendations, particularly for physical activity (for example, participants were tired after working at their own farms or had no resources for sports programmes). Furthermore, the geographic accessibility of sport programmes remained limited. This can explain the limited effectiveness of physical activity recommendations [46].

### Infobox 2 Questions for considering the usefulness of primary evidence for the target context [21]

- Is the investigated health problem clearly described?
- Is the study population described in detail, and is it relevant for the target context? (Is a similar target group addressed?)
- Is the intervention relevant for the health problem and/or goals in the target context?
- Are the outcomes (and measurements) relevant for the target context?
- Is the intervention up to date (still appropriate)?
- Are the effects of the intervention practically relevant?
- Are the descriptions of the intervention, environmental conditions, processes, and results sufficient to understand how the intervention works?
- Is the intervention potentially applicable to the target population/group(s) and setting(s)?
- Is the available evidence useful with regard to the level of transfer? (Has effectiveness of the intervention been demonstrated in a similar context?)
- Are documents and tools available (e.g., protocols or manuals for application and implementation)?

## Conclusion

The concept of transferability has gained in importance in Germany. An assessment of transferability is recommended before each implementation of an intervention. This is a complex task that comes with challenges. But it also offers opportunities: First, anticipating transferability can in the best case help in selecting and implementing a suitable and effective intervention in a target context. This can contribute to the careful use of limited resources. Second, considering transferability during the transfer process may facilitate understanding and reporting of the conditions under which an intervention works in a target context [21, 26]. On the basis of a differentiated data base, better causal models of the effectiveness of health promotion and disease prevention can be created in the long term [5]. This helps to establish an evidence base, which is practically relevant.

## Declarations

For this article no studies with human participants or animals were performed by any of the authors.
